# Cultural, Transcriptomic, and Proteomic Analyses of Water-Stressed Cells of Actinobacterial Strains Isolated from Compost: Ecological Implications in the Fed-Batch Composting Process

**DOI:** 10.1264/jsme2.ME15199

**Published:** 2016-05-28

**Authors:** Takashi Narihiro, Yuji Kanosue, Akira Hiraishi

**Affiliations:** 1 Department of Ecological Engineering, Toyohashi University of Technology ToyohashiAichi 441–8580Japan; 2 Bioproduction Research Institute, National Institute of Advanced Industrial Science and Technology (AIST)Tsukuba, Ibaraki 305–8566Japan; 3 Department of Environmental and Life Sciences, Toyohashi University of Technology ToyohashiAichi 441–8580Japan

**Keywords:** fed-batch composting, water activity, *Actinobacteria*, tetrazolium reduction, transcriptome, proteome

## Abstract

This study was undertaken to examine the effects of water activity (*a*_w_) on the viability of actinobacterial isolates from a fed-batch composting (FBC) process by comparing culturability and stainability with 5-cyano-2,3-ditoryl tetrazolium chloride (CTC). The FBC reactor as the source of these bacteria was operated with the daily loading of household biowaste for 70 d. During this period of composting, *a*_w_ in the reactor decreased linearly with time and reached approximately 0.95 at the end of operation. The plate counts of aerobic chemoorganotrophic bacteria were 3.2-fold higher than CTC-positive (CTC+) counts on average at the fully acclimated stage (after 7 weeks of operation), in which *Actinobacteria* predominated, as shown by lipoquinone profiling and cultivation methods. When the actinobacterial isolates from the FBC process were grown under *a*_w_ stress, no significant differences were observed in culturability among the cultures, whereas CTC stainability decreased with reductions in *a*_w_ levels. A cDNA microarray-based transcriptomic analysis of a representative isolate showed that many of the genes involved in cellular metabolism and genetic information processing were down-regulated by *a*_w_ stress. This result was fully supported by a proteomic analysis. The results of the present study suggest that, in low *a*_w_ mature compost, the metabolic activity of the community with *Actinobacteria* predominating is temporarily reduced to a level that hardly reacts with CTC; however, these bacteria are easily recoverable by exposure to a high *a*_w_ culture medium. This may be a plausible reason why acclimated FBC reactors in which *Actinobacteria* predominate yields higher plate counts than CTC+ counts.

Fed-batch composting (FBC) is a modification of conventional composting processes that is characterized by the repeated loading of biowaste without the removal of degraded and converted products. This process not only receives much attention for its practical applications, but also provides a potent model of microbial ecology in the biodegradation process of solid organic substances. A number of kinetic and microbiological studies have shown that the FBC process for the daily treatment of biowaste proceeds without four thermal stages comprising the mesophilic, thermophilic, cooling, and maturation phases (43), which are typically observed in the traditional batch composting process (14). Therefore, the FBC process for the treatment of biowaste has a lower and narrower temperature range that is favorable for the growth of mesophilic microorganisms. In a flowerpot-using FBC process (25) and commercially available FBC reactors (16, 41, 54) for garbage treatment, a population shift from members of the phylum *Proteobacteria* to those of the phylum *Actinobacteria* takes place during the start-up period of operation. Thus, the predominance of *Actinobacteria* under steady-state conditions is one of the most prominent biological features of the mesophilic FBC process. *Actinobacteria* are also common members of the microbiota in conventional composting systems, and constitute large parts of the community at the cooling and mature stages (14, 47, 50, 53, 56, 62).

Since FBC is operated without the removal of converted end products, mineral salts and non-biodegradable solutes from biowaste accumulate gradually with the operational time, thereby causing a decrease in water availability as defined by water activity (*a*_w_) or matric water potential (*ψ*_m_). Thus, *a*_w_ or *ψ*_m_ is a critical determinant of the physicochemical and biological characteristics of the FBC process (54) as well as those of conventional composting systems (35). In this relationship, it is important to note that none of the proteobacterial strains isolated from FBC reactors were unable to grow at *a*_w_ 0.970 and lower with high concentrations of polyethylene glycol 300 (PEG300) as the solute, whereas actinobacterial isolates grew at this low *a*_w_ level (54). This difference in growth responses to *a*_w_ stress between *Proteobacteria* and *Actinobacteria* is a possible reason why the latter group of bacteria becomes predominant in the FBC process under fully acclimated conditions (54).

There is a consensus that most prokaryotes in natural environments are not cultivable with laboratory-used culture media (4). On the other hand, the high culturability of bacteria in FBC reactors has been demonstrated by comparing plate counts to direct counts using epifluorescence microscopy with fluorochromes, SYBR Green, SYTO 9, and propidium iodide as the LIVE/DEAD *Bac*Light Bacterial Viability kit, and 5-cyano-2,3-ditoryl tetrazolium chloride (CTC) (40). One of the most interesting observations in this respect is that the CFU counts of bacteria were found to be markedly higher than CTC-stain-positive (CTC+) counts at the acclimated stage of FBC (54). This phenomenon is a puzzle because CTC assays for detecting metabolically active microorganisms are considered to cover the underestimation of viable microbes by plate counting. Significantly higher CTC+ counts than CFU counts have been detected in a wide variety of environments including drinking water (49) and activated sludge (59). The so-called “viable but non-culturable” bacteria (44) may be detected in part by CTC staining (36, 37, 45).

Therefore, the main purpose of this study was to evaluate the physiological state of *Actinobacteria* as the major population in the steady-state FBC process in order to obtain a plausible reason for why the process gives higher CFU counts than CTC+ counts. Information on the viability and metabolic activity of *Actinobacteria* in this process is important not only for understanding the mechanism underlying biodegradation during FBC, but also for improving process performance. In order to address this subject, we focused on the effects of *a*_w_ stress on the metabolic activity of actinobacterial isolates as studied by CTC staining and transcriptomic and proteomic analyses. Transcriptomic and/or proteomic approaches have increasingly been used to study the physiological responses of bacteria (10, 18, 19, 27, 30, 31, 39, 45) and fungi (61) to salt and water stresses. We herein demonstrate that *a*_w_ stress under growing conditions influenced *Actinobacteria* to reduce entire gene expression for energy metabolism and the resultant metabolic activity was at a level that hardly reacted with CTC; however, these *a*_w_-stressed cells are easily recoverable as CFU counts on ordinary culture media.

## Materials and Methods

### Operation of reactors and sample collection

A SANYO model SMS-K2 FBC reactor (SANYO Electric Co., Moriguchi, Japan), with a working volume of 32 L and containing 18 L of fresh wood chips (SANYO) as the solid matrix at the start of operation, was operated for 70 d at a waste-loading rate of 0.7 kg (wet wt) d^−1^. The biowaste used was food and kitchen waste collected from the restaurant of Toyohashi University of Technology. Detailed information on the structure of the reactor and the composition of the biowaste used has been described previously (41, 54). During the overall period of operation, the reactor was settled in an incubator room at 22°C and 50% humidity, and the temperature in the FBC reactor was not controlled, but changed spontaneously by self-heating. Solid waste and compost mixture (SCM) samples (5 g wet weight each) were collected from three different points of the core of the reactor at the end of each batch cycle. The samples were combined and screened by a stainless steel sieve with a 5-mm mesh in order to remove large pieces of waste. The samples were immediately subjected to physicochemical and microbiological analyses. Samples for the quinone analysis were stored at −30°C until used.

### Physicochemical analyses

Analyses of all physicochemical parameters were performed at the end of each batch cycle. The mass reduction rate by the reactor and electric conductivity, moisture content, and pH of the SCM were determined as described previously (41, 54). *a*_w_ was determined using an AW SPRINT HT-500 Water Activity analyzer (Novasina, Pfäffikon, Switzerland) according to the manufacturer’s instructions.

### Quinone analysis

Community succession in the FBC reactor was monitored using the biomarker method, quinone profiling (24). SCM samples were collected from the FBC reactor on d 5–70 (Table S1) and subjected to a quinone analysis. Colony-quinone profiling (40) was also performed for cultivable bacteria recovered as CFUs from the reactor as described below. Isoprenoid quinone were extracted with an organic solvent mixture, fractionated into menaquinone and ubiquinone fractions using a SepPak Vac cartridge (Waters Corporation, Milford, MA, USA), and separated by reverse-phase HPLC and photodiode array detection with external standards as described previously (23). Menaquinones, ubiquinones, and plastoquinones with *n* isoprene units in their side chain were abbreviated as MK-*n*, Q-*n*, and PQ-*n*, respectively. Partially hydrogenated ubiquinones and menaquinones were expressed as Q-*n*(H*_x_*) and MK-*n*(H*_x_*), respectively, where *x* indicated the number of hydrogen atoms saturating the side chain. Phylloquinone was abbreviated as K_1_. Pairwise differences in quinone profiles were evaluated using the dissimilarity index *D*, which is a modification of the city-block distance (22). Multi-dimensional scaling (MDS) (7, 32) of the *D* matrix data was performed using the XLSTAT program (Addinsoft, New York, NY, USA).

### Cell staining with fluorochromes and epifluorescence microscopy

SCM samples were suspended in filter-sterilized phosphate-buffered saline (PBS, pH 7.0), sonicated with 2-s intermittent bursts for 100 s (20 kHz; output power, 50 W), and diluted decimally with PBS for total cell counting and with 50 mM MOPS buffer (pH 6.5) for CTC+ cell counting. The total and viable counts of bacteria were directly measured by staining with SYBR Green I and with a LIVE/DEAD *Bac*Light Viability kit (Thermo Fisher Scientific, Waltham, MA, USA), respectively, as described previously (40). CTC staining to detect viable and metabolically active cells was performed according to the protocol described in (16), in which the reaction mixture was prepared to contain approximately 10^8^ cells mL^−1^. *Actinobacteria* and other possible Gram-positive bacteria among the CTC+ bacteria were specifically detected by a post-treatment with acetone (60). All stained specimens were observed under an Olympus model BX-50 epifluorescence microscope equipped with a DP-70 digital CCD camera (Olympus, Tokyo, Japan), and the number of stained cells was counted and analyzed using the WINROOF program (Flovel, Tachikawa, Japan).

### Enumeration, isolation, and identification of bacteria

Cultivable aerobic chemoorganotrophic bacteria in the reactor were enumerated using PBYG agar medium as reported previously (40, 54). Inoculated PBYG plates were incubated at 30°C for 2 weeks before the final counting of CFUs. Colonies on a “countable” plate were randomly selected for standard purification by streaking, whereas all colonies of other plates triplicated at the same dilution steps were harvested into test tubes for colony-quinone profiling as described above. The thirty strains isolated were subjected to PCR amplification and Sanger sequencing of 16S rRNA genes and quinone profiling as described previously (25, 54). The isolates were phylogenetically identified using the RDP Seqmatch search with the type and known strains as a data set option (8). Among the isolates identified, *Arthrobacter* sp. strain TUT3038 and *Rhodococcus* sp. strain TUT3051 were selected for further studies.

### Growth tests at different *a*_w_ levels

In addition to the two actinobacterial isolates described above, *Cellulosimicrobium* sp. strain TUT1222 and *Ornithinicoccus* sp. strain TUT1233, both of which were also isolated from an FBC reactor (42), were used to study physiological responses to *a*_w_ stress. The effects of *a*_w_ on the growth and physiological responses of bacteria have been studied in recent years using different solutes including NaCl (30), glycerol (51), and polyethylene glycol 8000 (18, 27). In this relationship, we preliminarily tested NaCl and the non-ionic solutes glycerol, ethylene glycol, and PEG300 for their effects on the growth of actinobacterial isolates. PEG300 was found to have the most severe effect on the growth of the test organisms among the four solutes at the same *a*_w_ level. Therefore, we used PEG300 to provide the required *a*_w_ levels in growth media through-out this study. As the growth medium, PBYG liquid medium was prepared to have different *a*_w_ levels by mixing with 0, 15, 25, and 30% PEG300 (w/v), which had *a*_w_ values of 0.999, 0.985, 0.968, and 0.957, respectively, as measured using the AW SPRINT HT-500 Water Activity analyzer (Fig. S1). The test organisms were grown aerobically at 30°C in screw-capped test tubes that contained different *a*_w_-containing media, and growth was monitored by measuring optical density at 660 nm (OD_660_) using a Shimadzu UV-1200 spectrophotometer (Shimadzu Corporation, Kyoto, Japan). Cultures at the late exponential phase of growth were sampled and tested for CFU recovery and CTC reductions as described above. Data were statistically analyzed using the Student’s *t*-test, and significant differences between different *a*_w_ levels were evaluated at *P* < 0.01.

### RNA extraction and cDNA synthesis

A representative of the FBC isolates, *Rhodococcus* sp. strain TUT3051, was grown aerobically at 30°C in PBYG medium supplemented with PEG300 to give *a*_w_ 0.957 as well as in PEG300-free medium as the control (*a*_w_ 0.999). Cells from cultures at the late exponential phase of growth, *i.e.*, 48-h-old culture at *a*_w_ 0.957 and 24-h-old culture at *a*_w_ 0.999 (see Fig. 5a), were harvested by centrifugation at 12,600×*g* for 10 min, washed with RNA*later* (Thermo Fisher Scientific), and resuspended in TE buffer (pH 8.0). RNA from these suspensions was extracted using a RiboPure RNA Purification Kit (Thermo Fisher Scientific) and then treated with DNase I according to the protocol accompanying the product. mRNA quality was checked by verifying intact 16S- and 23S-rRNA bands and quantifying the absorbance ratios at 260 to 280 nm and at 260 to 230 nm using the MICROARRAY function on a NanoDrop 2000 spectrophotometer (Thermo Fisher Scientific). cDNA synthesis was performed using 10 μg of total RNA, 1.25 μM of random hexanucleotide primers (Promega, Madison, WI, USA), 100 μM each of dATP, dGTP, dCTP, and dTTP, and 400 units of SuperScript II reverse transcriptase (Thermo Fisher Scientific) in a 50-μL scale according to a previously described protocol (55). cDNA was then labeled using a NimbleGen One-Color DNA Labeling Kit with Cy3-random nonamers, a dNTP mixture, and Klenow Fragment (lacking 3′–>5′ exonuclease activity) according to the manufacturer’s instructions (Roche Diagnostics). Labeled cDNA products were purified using the MinElute PCR purification kit (Qiagen, Venlo, Netherlands), and the quantities and incorporation efficiencies of Cy3-labeled NTPs were calculated using the MICROARRAY function on a NanoDrop 2000 spectrophotometer.

### Transcriptome analysis using microarrays

Transcriptomic assays were performed by GeneFrontier Corporation (Kashiwa, Japan) using Cy3-labeled cDNA and Roche NimbleGen ready-made microarray chips equipped with 3,426 genes from the whole genome of *Mycobacterium bovis* AF2122/97 (accession number NC_002945) (17). Microarray hybridization was performed according to a previously described protocol (55). The fluorescence signals of array chips were detected by scanning with a NimbleGen MS200 microarray scanner and NimbleGen Extinction software, and were normalized by using the Robst Multi-chip Average (RMA) algorithm (6). Normalized data were statistically analyzed by the Student’s *t*-test, and genes with expression levels that were significantly different between the two samples (*α*=0.05) were selected for further analyses. Information on genes and their products sampled by cDNA microarray analyses was obtained from the GenBank/EMBL/DDBJ DNA database. The functional classification of genes was performed by referring to the KEGG PATHWAY Database (http://www.genome.jp/kegg/pathway.html) and TubercuList (34).

### Protein extraction and two-dimensional electrophoresis

Cells of *Rhodococcus* sp. strain TUT3051 were grown aerobically at *a*_w_ 0.999 and 0.957 and harvested by centrifugation as described above. Cells were washed three times with 50 mM Tris-HCl (pH 7.5), re-suspended in Reagent 2 in the ReadyPrep Protein Extraction Kit (Bio-Rad Laboratories, Hercules, CA, USA), and sonicated three times for 1 min each on ice (20 kHz; output power, 150 W), followed by centrifugation at 20,000×*g* for 10 min. The resultant supernatants were saved, mixed with 4 volumes of cold acetone, and settled for 2 h at −20°C. The precipitate fraction was withdrawn by centrifugation, and protein clean-up was performed using a PlusOne 2D Clean-Up kit (GE Healthcare) according to the manufacturer’s instructions. The pellet obtained after the clean-up procedure was dissolved in Reagent 2 extraction buffer (Bio-Rad) containing one tablet of cOmplete, Mini Protease Inhibitor cocktail (Roche) in every 10 mL of extraction buffer and was then solubilized as described previously (26). The concentration of protein extracts was determined colorimetrically using the Protein Assay kit (Bio-Rad) according to the protocol accompanying the product. Extracted proteins (100 μg) were mixed with Reagent 2 extraction buffer (Bio- Rad) and analyzed by two-dimensional polyacrylamide gel electrophoresis (2D SDS-PAGE) using a Multiphor II Electrophoresis system (GE Healthcare, Little Chalfont, UK). First-dimensional isoelectric focusing was performed using an Immobiline DryStrip Kit (pH 4–7, 18 cm), and second dimensional separation was then conducted by sodium dodecyl sulfate-PAGE (SDS-PAGE) using ExcelGel XL SDS 12–14 (25 cm) according to the protocols described in (11) and specified by the manufacturer. Protein spots on the gels were detected by silver staining with the PlusOne Silver Staining Kit, Protein (GE Healthcare) according to the protocols specified by the manufacturer and modified for subsequent peptide mass fingerprinting (58). Gel images were scanned with an ImageScanner III system (GE Healthcare) and analyzed using the Image Master 2D version 3.0 program (GE Healthcare).

### Mass spectrometry and protein identification

Protein spots from 2D SDS-PAGE gels were excised, destained, in-gel digested with trypsin (Promega, Madison, USA), and extracted as described (11). Peptide mass fingerprinting was performed by the Pro Phoenix Division, Towa Environment Science Co. (Osaka, Japan) using a matrix-assisted laser desorption/ionization time-of-flight mass spectrometer (MALDI-TOF/MS) (Bruker Daltonics, Karlsruhe, Germany). Peptide sequence data were searched using the Mascot (Matrix Science, Boston, MA, USA) database search engine (http://www.matrixscience.com/) and the protein BLAST program (2) with the optional target organisms *Rhodococcus pyridinivorans* AK37 (taxid: 1114960), *Rhodococcus pyridinivorans* SB3094 (taxid: 1435356), and *Mycobacterium bovis* AF2122/97 (taxid: 233413). In some cases, a standard BLAST search without the optional sets was also performed. The average molecular weight (MW) of reference proteins based on amino acid sequences was predicted using the GENETYX-MAC version 17.0.7 program (GENETYX, Tokyo, Japan).

### Nucleotide database accession numbers

The 16S rRNA gene sequences of the actinobacterial strains used in this study have been deposited under DDBJ accession numbers AB188217 (*Cellulosimicrobium* sp. strain TUT1222), AB188219 (*Ornithinicoccus* sp. strain TUT1233), LC085892 (*Arthrobacter* sp. strain TUT3038), and LC085893 (*Rhodococcus* sp. strain TUT3051).

## Results and Discussion

### Changes in physicochemical parameters and bacterial counts

In order to confirm the characteristic features of the FBC process previously described (16, 41, 54), we monitored physicochemical and microbiological parameters in this process during 70 d of composting. After 6 weeks of operation, the FBC reactor had a core temperature of 32–40°C, pH 9.5–9.7, and moisture content of 33–40%. *a*_w_ decreased linearly with time, and reached the lowest level of approximately 0.950 on d 70 while highly fluctuating after 6 weeks of oper ation (Fig. S2a). Electric conductivity increased linearly with time and reached 4.1 mS cm^−1^ with high fluctuations in the fully acclimated stage. These FBC data were consistent with those previously reported (16, 41, 54). The reverse relationship between *a*_w_ and conductivity may result from the time-dependent accumulation of minerals and other non-degradable solutes and a decrease in moisture content in the reactor. The electric conductivity level recorded at the end of operation (4.1 mS cm^−1^) corresponded to approximately 0.2% NaCl (w/v), and this ionic solute concentration was too low to reach an *a*_w_ level of 0.950 (Fig. S1). Therefore, the accumulation of non-ionic solutes may contribute more to decreases in *a*_w_ during the FBC operation.

All viable bacterial counts by the three different methods greatly fluctuated during the first 3 weeks of operation, with the highest counts being recorded on d 5, and gradually steadied thereafter (Fig. S2b). At the fully acclimated stage of operation (on d 35 and thereafter), the ratio of CFU counts to direct total counts was 50 ± 2% on average, which was consistent with our previous finding that FBC bacteria are highly culturable (40). At this stage, the CFU count was similar to the LIVE/DEAD count and was 3.2-fold higher than the CTC+ count (16 ± 2% of the total count) on average, thereby confirming previous findings on the relationship between CFU and CTC+ counts (54).

**Fig. 1:**
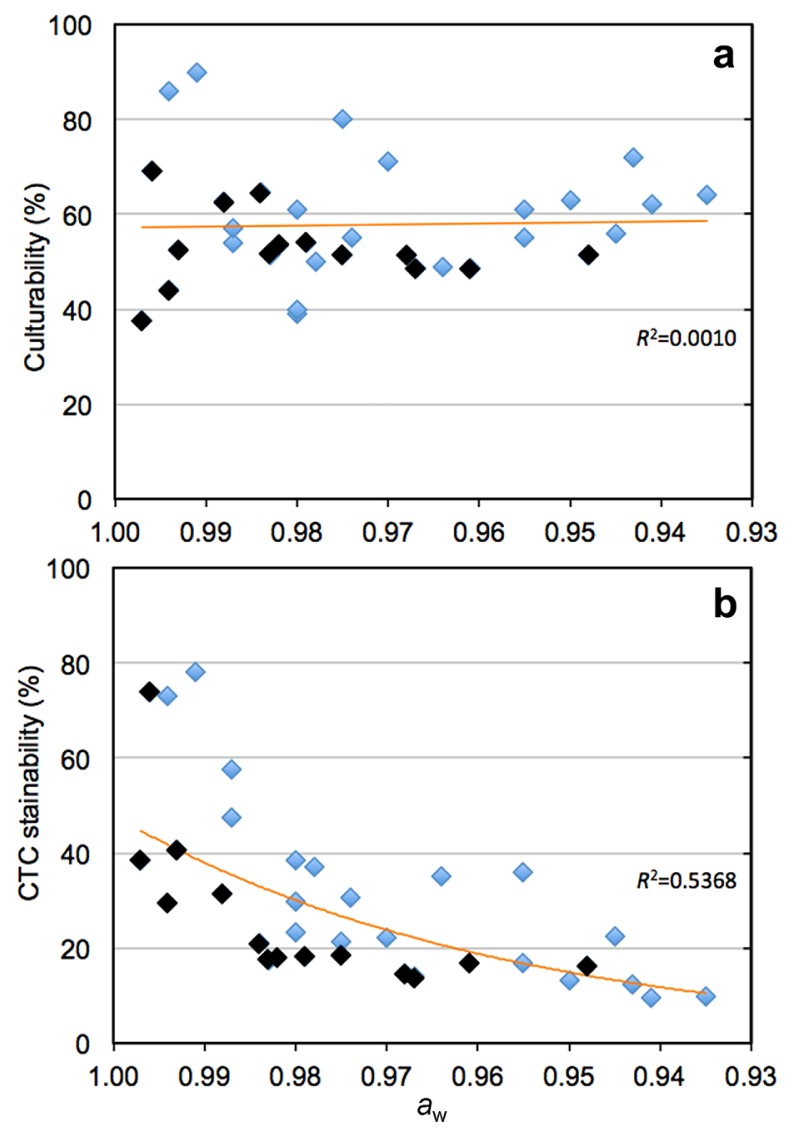
Relationship between the culturability (a) or CTC stainability (b) of bacteria and *a*_w_ levels during the FBC operation. The data obtained in the present study and previously (16, 54) are plotted and shown by black and blue diamonds, respectively. A correlation was observed between CTC stainability and *a*_w_ (*R*^2^=0.5368) (*P* < 0.01).

Using all data obtained in this study and previous findings (16, 54), we examined the relationship between the culturability and CTC stainability of bacteria and *a*_w_ levels (Fig. 1). The plotted data clearly showed that while % culturability did not correlate with *a*_w_ levels (Fig. 1a), a negative correlation (*P* < 0.01) was observed between % CTC stainability and *a*_w_ (Fig. 1b). As an example, CTC stainability in the FBC reactor on d 5 (*a*_w_ 0.996) and 63 (*a*_w_ 0.959) is shown in Fig. S3. Most CTC+ cells (>90%) on d 5 were decolorized with acetone, whereas almost all CTC+ cells on d 63 were not (data not shown). This result suggests that the predominant CTC+ bacteria at the acclimated stage were Gram-positive bacteria, among which CTC+ cells were resistant to the acetone treatment (60).

### Succession of microbial community by quinone profiling

A number of studies have demonstrated that isoprenoid quinones are good biomarkers of microbial populations in the environment in terms of quantity, quality, and activity (22–24). Thus, we monitored microbial community dynamics during the overall period of the FBC operation by quinone profiling.

The quinone analysis of SCM samples from the FBC reactor revealed that Q-8, Q-9, and Q-10 were the major quinone components detected during the first half of operation; however, partially saturated menaquinones, particularly MK-8(H_4_), increased significantly at the acclimated stage (Fig. S4a). These results clearly indicated microbial succession from ubiquinone-containing *Proteobacteria* to *Actinobacteria* as the major constituents of the microbiota during the FBC operation. An MDS analysis of pairwise *D* matrix data demonstrated this microbial succession, and suggested that the community structure converged after 34 d of operation (Fig. S4b). The obtained Kruskal stress value (0.068) indicated that the significance of this MDS analysis was fairly good (32).

When quinone profile data were plotted against the *a*_w_ level, the relative and absolute amounts of the MK-*n*(H*_x_*) fractions showed strong correlations with *a*_w_ (*P* < 0.01) (Fig. S5), thereby confirming our previous findings that *a*_w_ is a critical determinant of the predominance of *Actinobacteria* in the FBC process (16, 54). Furthermore, the relative content (mol%) of the Q-*n* fraction exhibited a positive correlation with the *a*_w_ level (*P* < 0.01) (Fig. S5a), whereas no correlation was found between the actual amount of the Q-*n* fraction and *a*_w_ (Fig. S5b). These results may be explained as follows; while the population density of ubiquinone-containing *Proteobacteria* in the FBC reactor was relatively constant (Fig. S4a), possibly by continuous seeding from the biowaste (54), their relative abundance decreased with increases in the populations of *Actinobacteria* under low *a*_w_ conditions.

Our concurrent study on FBC community dynamics based on the 16S rRNA gene-targeted clone library and Illumina MiSeq-using amplicon analyses did not give high data reproducibility or always produced results in which the proportion of actinobacterial clones (<40% of the total) was markedly smaller (<40% of the total) than expected from the MK-*n*(H*_x_*) content (unpublished data). A previous study on the FBC community also failed to detect the 16S rRNA gene clones of *Actinobacteria* by PCR-aided denaturation gradient gel electrophoresis (41). These results may be due to biases in PCR and/or DNA extraction from SCM samples. It is important to note that the reproducibility of the quinone analysis was higher than that of these molecular approaches and also had the advantage of providing quantitative and qualitative data on microbial biomass (Fig. S4a).

### Cultivation-based community analysis

**Fig. 2:**
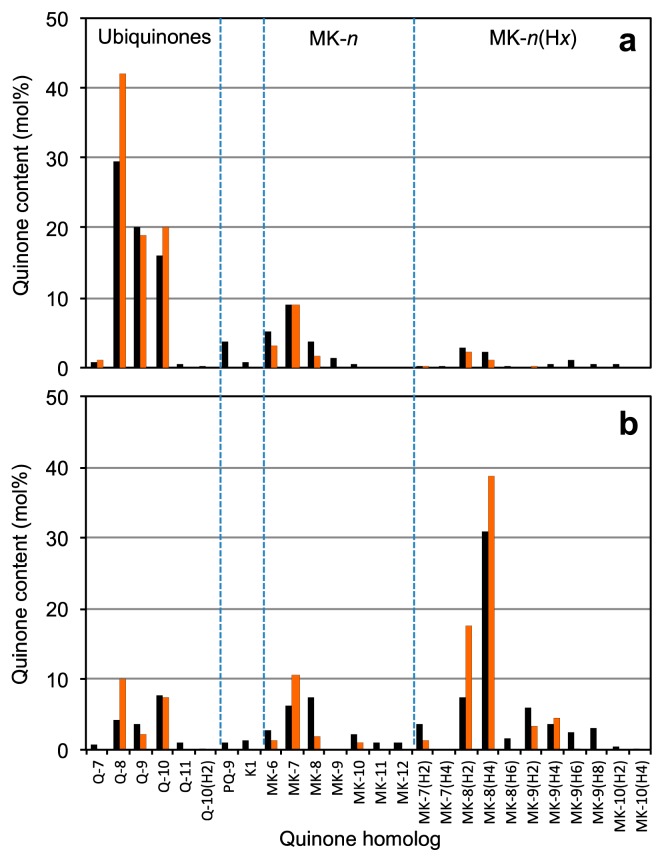
Comparative quinone profiles of SCM samples and bacterial colonies recovered by plating from them taken on d 5 (a) and 63 (b). The black and orange histograms show the data on SCM and colonies, respectively.

We also used a cultivation-based approach to the bacterial community analysis of the FBC reactor. Comparative quinone analyses of bacterial colonies recovered by plate counting and the SCM samples as their source showed that both quinone profiles were similar to one another (Fig. 2), suggesting that the cultivation methods worked well for the microbial community analysis of the FBC process. These results appear to be due to the high culturability of FBC bacteria (Fig. 1a and Fig. S2b), which is in accordance with our previous findings (40).

Thirty strains of aerobic chemoorganotrophic bacteria from the reactor on d 63 were isolated and classified into 11 operational taxonomic units (OTUs) on the basis of 16S rRNA gene sequencing and quinone systems (Table S1). Nineteen out of the 30 isolates (63%) were assigned with members of *Actinobacteria*. Among these isolates and those previously isolated from an FBC reactor (42), we selected four isolates: *Arthrobacter* sp. strain TUT3038, *Cellulosimicrobium* sp. strain TUT1222, *Ornithinicoccus* sp. strain TUT1233, and *Rhodococcus* sp. strain TUT3051, for further studies as described below. RDP Seqmatch searches with the type strains as a data set option showed that strains TUT1222, TUT1233, TUT3038, and TUT3051 were the most closely related to *Cellulosimicrobium funkei* strain W6122^T^ (accession number AY501364, 98% similarity), *Ornithinimicrobium murale* strain 01-Gi-040^T^ (FR874098, 93%), *Arthrobacter soli* strain SYB2^T^ (EF660748, 98%), and *Rhodococcus pyridinivorans* strain PDB9^T^ (AF173005, 98%), respectively.

### Physiological responses to different *a*_w_ levels of isolates

**Fig. 3:**
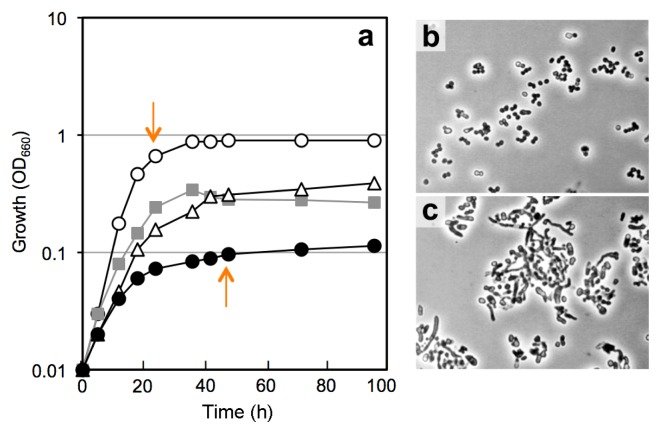
Growth and cell morphology of *Rhodococcus* sp. strain TUT3051 at different *a*_w_ levels. Symbols in (a): open circles, *a*_w_ 0.999; grey squares, *a*_w_ 0.982, open triangles, *a*_w_ 0.974; closed circles, *a*_w_ 0.957. Fig. 3b and 3c show phase-contrast micrographs of cells grown at *a*_w_ 0.999 and 0.957, respectively; cultures for microscopic studies were sampled at the late exponential phase of growth (shown by arrows in Fig. 3a). Similar results were obtained with the other 3 test strains of *Actinobacteria*.

**Fig. 4:**
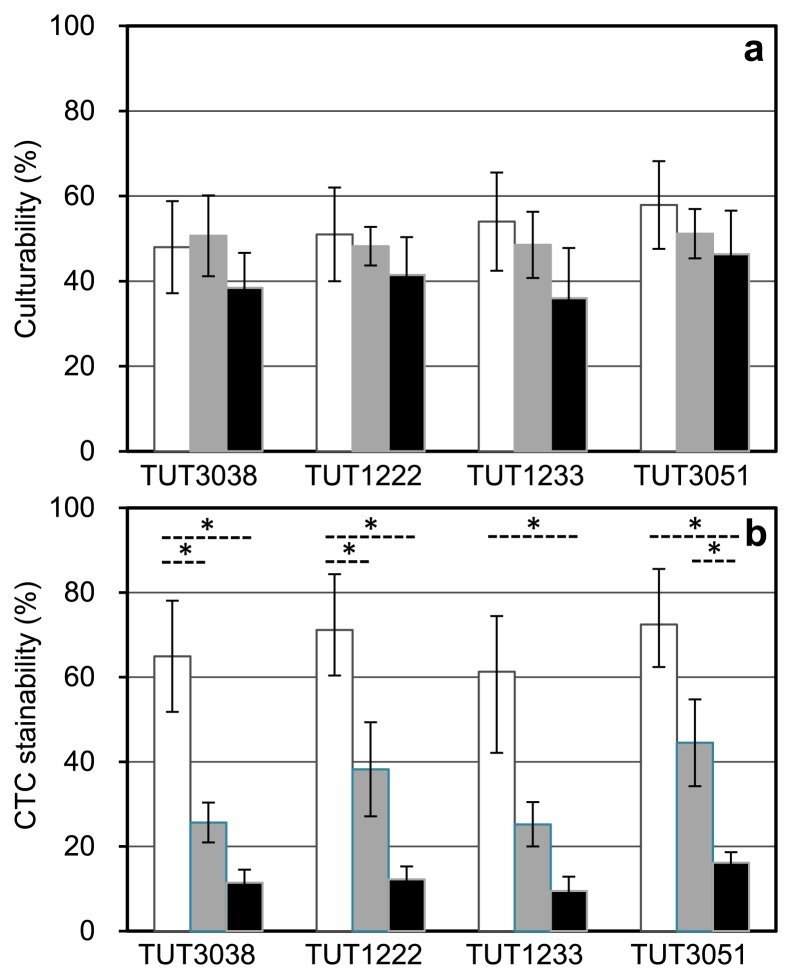
Culturability (a) and CTC stainability (b) in cultures of *Arthrobacter* sp. strain TUT3038, *Cellulosimicrobium* sp. strain TUT1222, *Ornithinicoccus* sp. strain TUT1233, and *Rhodococcus* sp. strain TUT3051 grown at different *a*_w_ levels. Open, gray, and black histograms show data on *a*_w_ 0.999, 0.982, and 0.974, respectively. Cultures at the late exponential phase of growth were sampled and subjected to plate counting and CTC staining. Data show averages and standard deviations of three different determinations. Asterisks show that differences between the samples are significant (*P* < 0.01).

In order to clarify why the culturability of bacteria is markedly higher than expected from CTC+ counts and the population of *Actinobacteria* rather than that of *Proteobacteria* increases in the steadied FBC reactor, we examined the effects of *a*_w_ stress on the growth and viability of the selected actinobacterial isolates. The four test strains, *Arthrobacter* sp. TUT3038, *Cellulosimicrobium* sp. TUT1222, *Ornithinicoccus* sp. TUT1233, and *Rhodococcus* sp. TUT3051, were cultivated in PBYG broth having an *a*_w_ level of 0.999–0.957 with different concentrations of PEG300, and culturability and CTC stainability as well as growth responses were investigated. Data for a representative actinobacterium, strain TUT3051, is shown in Fig. 3. *Rhodococcus* sp. TUT3051 and all other test organisms grew more weakly and slowly in response to lower *a*_w_ levels up to *a*_w_ 0.957 (Fig. 3a), at which proteobacterial isolates were unable to grow, as reported previously (54). The test isolates showed an elongated, unusual cell morphology at *a*_w_ 0.957 (cf. Fig. 3b and 3c), suggesting that cell division was inhibited by low *a*_w_. The CFU recovery rate by plating from late-exponential cultures decreased slightly with reductions in *a*_w_ levels in all test organisms (Fig. 4a); however, these differences in culturability were not significantly different. On the other hand, significant differences in CTC stainability among different *a*_w_ levels were observed in all cases (*P* < 0.01). CTC+ counts decreased more than CFU counts in response to *a*_w_ changes (Fig. 4b).

Morphological changes in response to reduced *a*_w_ levels, *i.e.*, the formation of elongated cells, have been reported in *Bacillus subtilis* strain 168^T^ (9). Furthermore, salt stress during the growth of *Desulfovibrio vulgaris* strain Hildenborough^T^ resulted in growth-rate reductions and cell elongation through the inhibition of DNA replication (39). With respect to water stress-responding tetrazolium reduction, Whiteley *et al.* (57) showed that the CTC+ count decreased more in dried soil microcosms than in wetted microcosms. Ponder *et al.* (46) reported that the CTC-reducing activities of *Psychrobacter arcticus* and *Exiguobacterium sibiricum* isolates as well as that of *Escherichia coli* strain B decreased in response to osmotic stress. Regarding the survival of *Rhodococcus opacus* strain PD630 under water-stressed conditions, metabolic activity as measured by a tetrazolium reduction assay decreased more rapidly than the colony recovery rate (3). In view of these findings together with our results, it is likely that *a*_w_ stress during growth affects *Actinobacteria* by not only repressing cell division, but also inducing a metabolically inactive state such that they hardly react with CTC. Meanwhile, the water-stressed cells of *Actinobacteria* may restore normal growth and morphogenesis if they are exposed once to such high *a*_w_ environments as culture media.

### Transcriptomic analysis by cDNA microarray

**Fig. 5:**
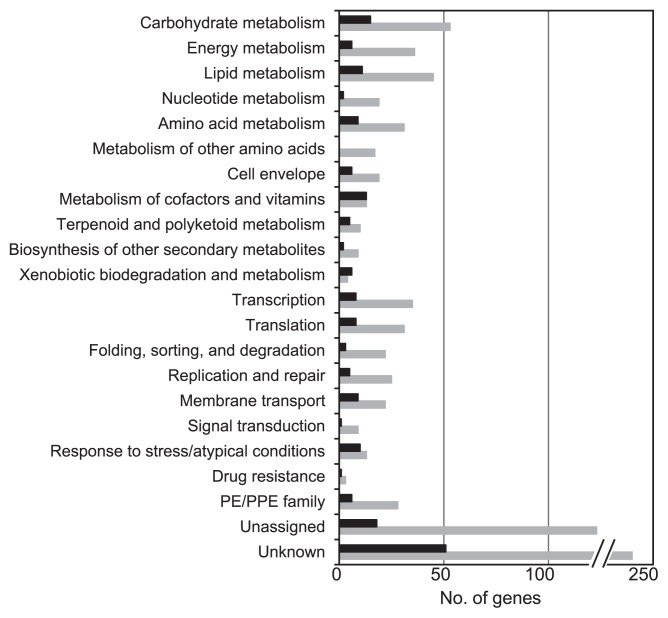
Functional classification of *Rhodococcus* sp. strain TUT3051 genes showing significant differences in transcription in response to *a*_w_ stress. The black and grey histograms show up-regulated and down-regulated genes, respectively.

In order to study the effects of *a*_w_ stress on the metabolic activity of *Actinobacteria* in more detail, we performed transcriptomic assays using cultures of a representative isolate, *Rhodococcus* sp. strain TUT3051. Cells were grown at *a*_w_ 0.957 and 0.999 (as the control), processed for RNA, and subjected to a cDNA microarray analysis with a set of the *Mycobacterium bovis* AF2122/97 genes. The fluorescence microarray signals thus detected and normalized using the RMA algorithm (6) were shifted entirely to low levels by scatter plotting because of cross hybridization between the phylogenetically related, but taxonomically different species (Fig. S6). Nevertheless, 1,005 genes with expression levels that were significantly different between the two different *a*_w_ cultures were detected (α=0.05). Of the 1,005 genes thus obtained, 198 (19.8%) were differentially expressed in the *a*_w_ 0.957 culture and 807 (80.2%) in the control culture (Table 1). The functional classification of the sampled genes is summarized in Fig. 5, and selected genes that showed a transcriptional fold change of more than 1.5 are listed in Table S2.

### Genes down-regulated by *a*_w_ stress

As shown in Fig. 5, the low *a*_w_, 0.957, exerted stress on the *Rhodococcus* isolate to down-regulate genes in most of the functional categories. Since the CTC stainability of cells depends on redox activities in cellular and energy metabolism, it is important to note that down-regulation occurred in a number of genes that participate in respiratory electron transport and dehydrogenation in glycolysis-related pathways and the TCA cycle. These included *gap* (glyceraldehyde-3-phosphate dehydrogenase), *adhE2* (zinc-dependent alcohol dehydrogenase), *icd1* (isocitrate dehydrogenase), *sdhC* (succinate dehydrogenase cytochrome B-556 subunit), *ndh* (NADH dehydrogenase), *nuoM* (NADH dehydrogenase subunit M), *cydB* (integral membrane cytochrome D ubiquinol oxidase CydB), and *ctaE* (cytochrome C oxidase subunit III) (Table S2). Furthermore, many of the genes that are responsible for genetic information processing were down-regulated. These included *dinX* (DNA polymerase IV), *sigL* (RNA polymerase sigma factor SigL), *rpoB* (DNA-directed RNA polymerase beta subunit), and a number of transcriptional regulators and ribosomal proteins (Table S2). In addition, down-regulation was detected in cell proliferation-associated genes, *e.g.*, the *fts* genes (DNA translocase FtsK, cell division protein FtsX, and cell division protein FtsZ) and *prfb* (peptide chain release factor 2) (1.2- to 1.6-fold, data not shown and partly in Table S2), and molecular chaperone genes, *dnaJ1* (heat shock protein 70) and *dnaK* (heat shock protein 40).

Similar results for transcriptomes have been reported in the stressed cells of some members of *Proteobacteria*. In *Shewanella algae* strain 2736, the genes encoding glyceraldehyde 3-phosphate dehydrogenase and alcohol dehydrogenase were down-regulated at the transcriptional level at 1 h after salt stress (15). *E. coli* strain K-12 down-regulated genes that participate in energy metabolism and cell proliferation under continuous osmotic and heat stresses (19). *Pseudomonas putida* strain KT2440 down-regulated many of the genes involved in energy metabolism and encoding heat shock proteins in response to water stress (18). Taken together, our transcriptomic data provide a possible explanation for why the tested actinobacterial strains grew weakly with unusual morphogenesis at *a*_w_ 0.957 (Fig. 3c) and the CTC stainability of growing cells decreased with reductions in *a*_w_ levels (Fig. 4b).

### Genes up-regulated by *a*_w_ stress

Among the smaller numbers of genes up-regulated by *a*_w_ stress, a higher fold change was found in *dnaB* (Table S2), which encodes replicative DNA helicase, involved in the formation of the DNA replication fork and DNA melting (29). Similar results were obtained with *Shewanella algae* strain 2736, in which DNA helicase genes were up-regulated after salt stress (15). Since salt stress affects the stability of nucleic acid base pairing, the transcriptional up-regulation of *dnaB* under *a*_w_ stress may be a response to maintain genetic information processing, as suggested previously (39). Furthermore, genes up-regulated at *a*_w_ 0.957 (≥ 1.5-fold) included PE/PEE family genes, *ponA1* (bifunctional penicillin-binding protein), and several other functionally unknown genes (Table S2). Previous studies demonstrated that PE/PEE family protein genes, encoding functionally unknown membrane proteins in mycobacteria, are expressed at the transcript level in response to starvation (5), heat shock (52), and acidic conditions (13). The *ponA1* gene is involved in cell wall formation (peptidoglycan synthesis) in *Mycobacterium tuberculosis* strain H37Rv^T^, and its increased expression was detected under anaerobic non-replicating conditions (48).

In addition to the aforementioned results, the genes involved in the metabolism of co-factors and vitamins were up-regulated in response to *a*_w_ stress. The number of differentially expressed genes in this functional category was similar between the two *a*_w_ cultures. *a*_w_ stress-induced genes included *thiL* (thiamine-monophosphate kinase THIL), *nibF* (bifunctional riboflavin kinase), and *moaA* (molybdenum cofactor biosynthesis protein A) (Table S2). Genes that participate in cellular defenses against oxidative stress and damage also responded to *a*_w_ stress. These were represented by the thioredoxin-encoding gene, *trxA* (Table S1), *nrdH* (glutaredoxin), *sodA* (superoxide dismutase [Fe] SodA), and *sodC* (superoxide dismutase [Cu-Zn] SodC) (1.2- to 1.4-fold, data not shown). Akif *et al.* (1) suggested that multiple thioredoxins in *M. tuberculosis* strain H37Rv^T^ provide a cover for coping with metabolic demands and oxidative stress. Gunasekera *et al.* (19) found that a number of genes involved in defenses against oxidative damage in *E. coli* strain K-12 were up-regulated under continuous osmotic and/or heat stresses.

A previous study reported that *E. coli* induced genes encoding osmoprotectant proteins, *e.g.*, the genes of the *proU* operon as well as oxidative stress-responsive genes, when grown in the presence of 0.3 M NaCl (19). However, in the present study, the genes that encode the osmoprotectant (glycine betaine/L-praline) transport integral membrane proteins, *prow*, *prove*, *proxy*, and *prose*, were down-regulated (1.3- to 1.5-fold) in the *a*_w_ 0.957 culture. We used the non-permeable solute PEG300 to provide the required *a*_w_ levels in culture media, and this may be a possible reason for why the genes of the *proU* operon were not up-regulated in our low *a*_w_ culture. A transcriptome study of *Sphingomonas wittichii* strain RW1^T^ revealed that, even though permeating and non-permeating solutes have similar effects on growth rates, these solutes affect cells in fundamentally different ways (27). Therefore, it is necessary to investigate the effects of ionic solutes on the metabolic activity and transcriptomes of actinobacterial isolates in more detail.

### Proteome in response to water activity stress

As noted above, a limitation to our microarray analysis is that data resulted from cross hybridization between taxonomically different species. Scatter plotting of microarray signals from the two experiments showed an entire shift to lower levels (Fig. S6) than those with the usual cDNA-DNA hybrids of the same species origin. In order to complement the microarray data, we performed proteomic analyses on *Rhodococcus* sp. strain TUT3051 grown under the same conditions as those for the transcriptome.

**Fig. 6:**
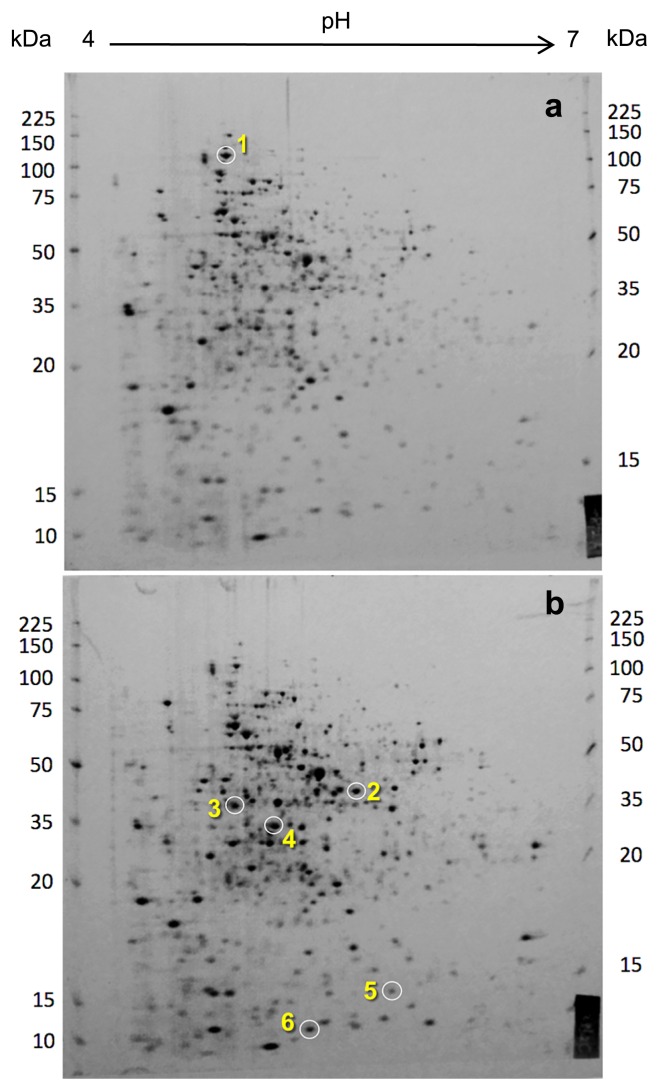
2D SDS-PAGE of whole proteins extracted from *Rhodococcus* sp. strain TUT3051 grown at *a*_w_ 0.957 (a) and 0.999 (b). Differentially appearing spots designated as nos. 1–6 were identifiable by TOF-MS analyses.

The protein spot profiles from the two cultures by 2D SDS-PAGE and silver staining were similar to each other in appearance (Fig. 6). However, the number of protein spots detected by the image analysis were 780 in the *a*_w_ 0.957 culture and 865 in the *a*_w_ 0.999 culture, indicating a 10% decrease in protein numbers in the former culture. These results are consistent with transcriptional data that show markedly smaller numbers of differentially expressed genes in the low *a*_w_ culture than in the control (Table 1).

Among a number of differentially expressed proteins, we excised more than 20 spots from both gels and identified them by mass fingerprinting. Based on the apparent molecular size on the gel, the determined peptide sequences, and results of the cross-homology search, we identified only one excised sample from the *a*_w_ 0.957 sample (Fig. 6a) and 5 from the control (Fig. 6b). These were spot 1 protein (110 kDa) as a DNA helicase, spot 2 (40 kDa) as a *Gordonia* multispecies zinc-containing alcohol dehydrogenase (corresponding to AdhB in *M. bovis* AF2122/97), spot 3 (38 kDa) as an alcohol dehydrogenase (corresponding to AdhE2 in *M. bovis* AF2122/97), spot 4 (30 kDa) as a *Rhodococcus* multispecies hypothetical protein (corresponding to Wag31 in *M. bovis* AF2122/97), spot 5 (14 kDa) as a *Rhodococcus* multispecies hypothetical protein, and spot 6 (9 kDa) as ribosomal protein L29 (Table S3).

Although the number of proteins actually identified by peptide sequencing was small, the proteome data obtained were in agreement with the results of cDNA microarray assays, which showed the up-regulation of *dnaB* and down-regulation of *adhB*, *adhE2*, and *wag31* by *a*_w_ stress (Table S2). Furthermore, the *rpmC* gene, encoding ribosomal protein L29, was down-regulated by 1.3-fold (data not shown). Wag31, first identified as a mycobacterial antigen (21), has been suggested to regulate cell division and cell shape by its appearance on the cell surface (28) and also to play a role in protection from oxidative stress (38) in mycobacteria. Therefore, the unusual morphogenesis of strain TUT3051 cells grown at *a*_w_ 0.957 (Fig. 3c) may be related to these repressed and expressed membrane-bound proteins (*i.e.*, Wag31 and PonA1).

### Physiological state of *Actinobacteria* in water-stressed FBC

While the survivability of microorganisms in natural environments depends on a number of physiological and genetic factors (20), *Actinobacteria* are known to be protected from desiccation by features inherent to Gram-positive cell walls and are able to persist longer under low *a*_w_ conditions (51) than Gram-negative bacteria. These features of *Actinobacteria* may also be achieved by presumably beneficial genetic responses to water stress (33). By these collective data and our previous findings and results described herein (54), it is logical to conclude that *Actinobacteria* preferentially inhabit the FBC process over *Proteobacteria* under steady-state conditions, which has low moisture and *a*_w_ levels.

As described in the present study, the cultivation of the *Rhodococcus* isolate under low *a*_w_ conditions resulted in a decrease in entire gene expression associated with dehydrogenation in glycolysis and the TCA cycle, respiratory electron transport, and chromosomal replication, and an increase in the expression of gene functions involved in DNA repair, the biosynthesis of cofactors and vitamins, and cellular defenses against oxidative stress and damage. These results suggest that although *a*_w_ stress exerts pressure on the actinobacterium to reduce the functions of cellular redox reactions and replication, it may still maintain viability under *a*_w_ stress by tuning the gene expression of a wide variety of cellular processes and defense systems. Similar findings have been reported in starvation-stressed *Sphingomonas* strain LH128, which shows a number of cellular and genetic adaptive strategies to survive during a prolonged period of starvation without a marked loss in viability (12).

The procedure for CTC staining used in the present study runs for 4 h (16), and this time may be insufficient for *a*_w_-stressed cells to restore normal gene expression and the resultant metabolic activity needed to fully react with CTC. However, these inactive cells may display normal growth when exposed for a longer time to plate-counting media. This is a plausible reason for why CFU counts were higher than CTC+ counts in the acclimated FBC process as well as under *a*_w_-stressed cultures. Our result that *a*_w_ and electric conductivity values fluctuated highly during the steady-state FBC operation (Fig. S2a) suggests that an inhabitable environment for *Actinobacteria* is given on the interface between mature compost and daily loaded biowaste in the steadied FBC reactor. Specifically, the FBC community with *Actinobacteria* predominating is inactive in low *a*_w_ mature compost, but activity is restored once it is exposed to high *a*_w_ biowaste (Fig. S7).

The conditions of cultivation with laboratory-used media may hardly mimic those in natural microbial habitats. However, in view of bacteria in the FBC reactor being highly culturable, as reported here and previously (40), our results on the isolate grown under laboratory conditions are significant for assessing the physiological state of *Actinobacteria* during the FBC operation. In order to obtain more definite information, more strains of *Actinobacteria* and *in situ* FBC communities need to be examined from physiological, transcriptomic, and proteomic viewpoints.

## Conclusions

The present study confirmed that *a*_w_ linearly decreases with time during mesophilic FBC, and this process yields higher CFU counts with *Actinobacteria* predominating than CTC+ counts under steady-state conditions. Low *a*_w_ levels influence *Actinobacteria* to reduce entire gene expression associated with cellular redox processes and CTC-reducing activity; however, this stressed state of the organisms may be immediately restored by exposure to high *a*_w_ conditions.

## Supplementary Material



## Figures and Tables

**Table 1:** Number of genes with expression levels that were significantly different between two different *a*_w_ cultures of *Rhodococcus* sp. strain TUT3051

Fold change in comparison	No. of genes differentially expressed at:^a^	

	*a*_w_ 0.957	*a*_w_ 0.999
8.0 ≤	0	0
4.0–<8.0	0	2
2.0–< 4.0	2	94
1.5–< 2.0	21	287
1.5>	175	424
Total	198	807

a Difference evaluated by the Student’s *t*-test (α = 0.05).
