# Supplementary material for: Cultural, Transcriptomic, and Proteomic Analyses of Water-Stressed Cells of Actinobacterial Strains Isolated from Compost: Ecological Implications in the Fed-Batch Composting Process
Source: Microbes Environ. 2016 May 28;31(2):127–36. doi: 10.1264/jsme2.ME15199 (PMC4912147; doi:10.1264/jsme2.ME15199)
Supplement: Supplementary file 1 [file 31_127_s1.pdf]

Supplementary Material

Cultural, Trasncriptomic, and Proteomic Analyses of Water Stressed Cells of Actinobacterial Strains Isolated from Compost: Ecological Implications in the Fed-Batch Composting Process

Takashi Narihiro[1,2], Yuji Kanosue[1], and Akira Hiraishi[1,3*]

[1]*Department of Ecological and Engineering, Toyohashi University of Technology Toyohashi, Aichi 441-8580, Japan*; [2]*Bioproduction Research Institute, National Institute of Advanced Industrial Science and Technology (AIST), Tsukuba, Ibaraki 305-8566, Japan*; *and* [3]*Department of Environmental and Life Sciences, Toyohashi University of Technology Toyohashi, Aichi 441-8580, Japan*

*Corresponding author. E-mail: hiraishi@ens.tut.ac.jp; Tel: +81 532 44 6913; Fax: +81 532 44 6929.

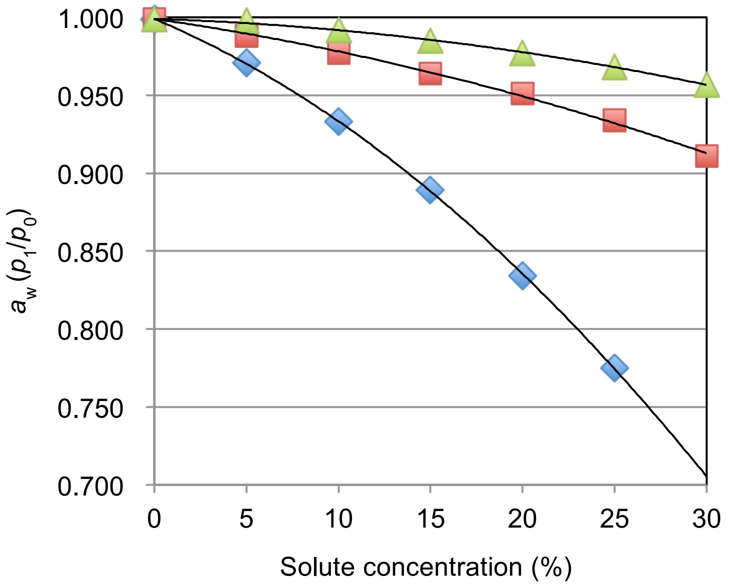

Fig. S1. Water activity of PBYG medium containing different concentrations of solutes. Symbols: diamonds, NaCl; squares, glycerol; triangles, PEG300. Water activity was measured using an AW SPRINT HT-500 Water Activity analyzer at 25°C.

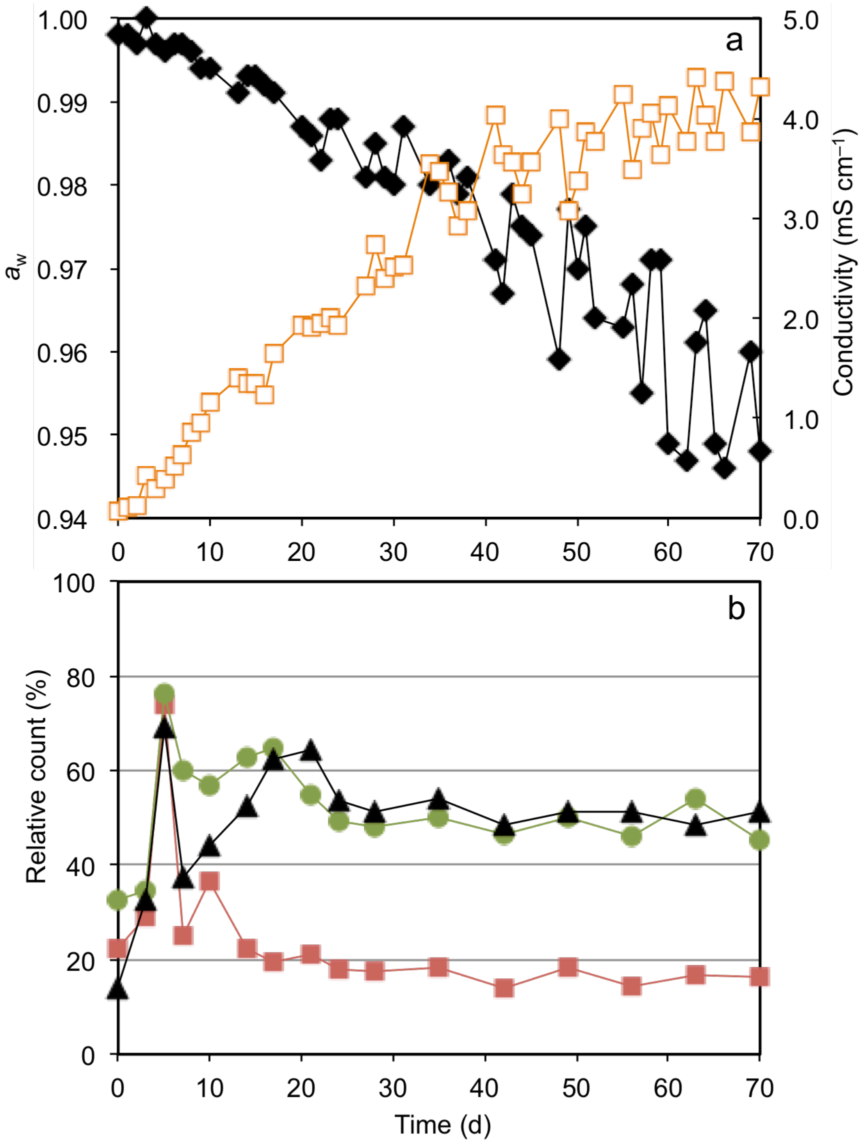

Fig. S2. Changes in a_w, electric conductivity, and relative bacterial counts during the overall period of FBC operation. Symbols in (a): closed diamonds, a_w; open squares, conductivity. Symbols in (b): circles, total viable counts by the LIVE/DEAD kit; squares, CTC+ counts; triangles, CFU counts; all these counts are shown as % of the direct total counts. The direct total counts were 1.5–3.0×10^{11} g^{-1} (wet wt) at the fully acclimated stage (on days 35–70).

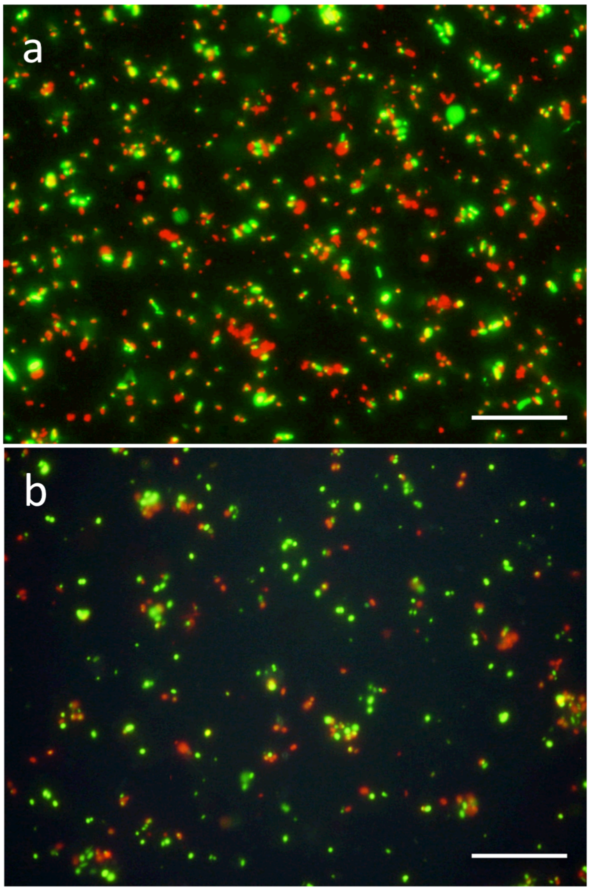

Fig. S3. Epifluorescence micrographs (merged images) of SYBR-Green- and CTC-stained microorganisms in the FBC reactor on days 5 (a) and 63 (b). Scale = 20 μm.

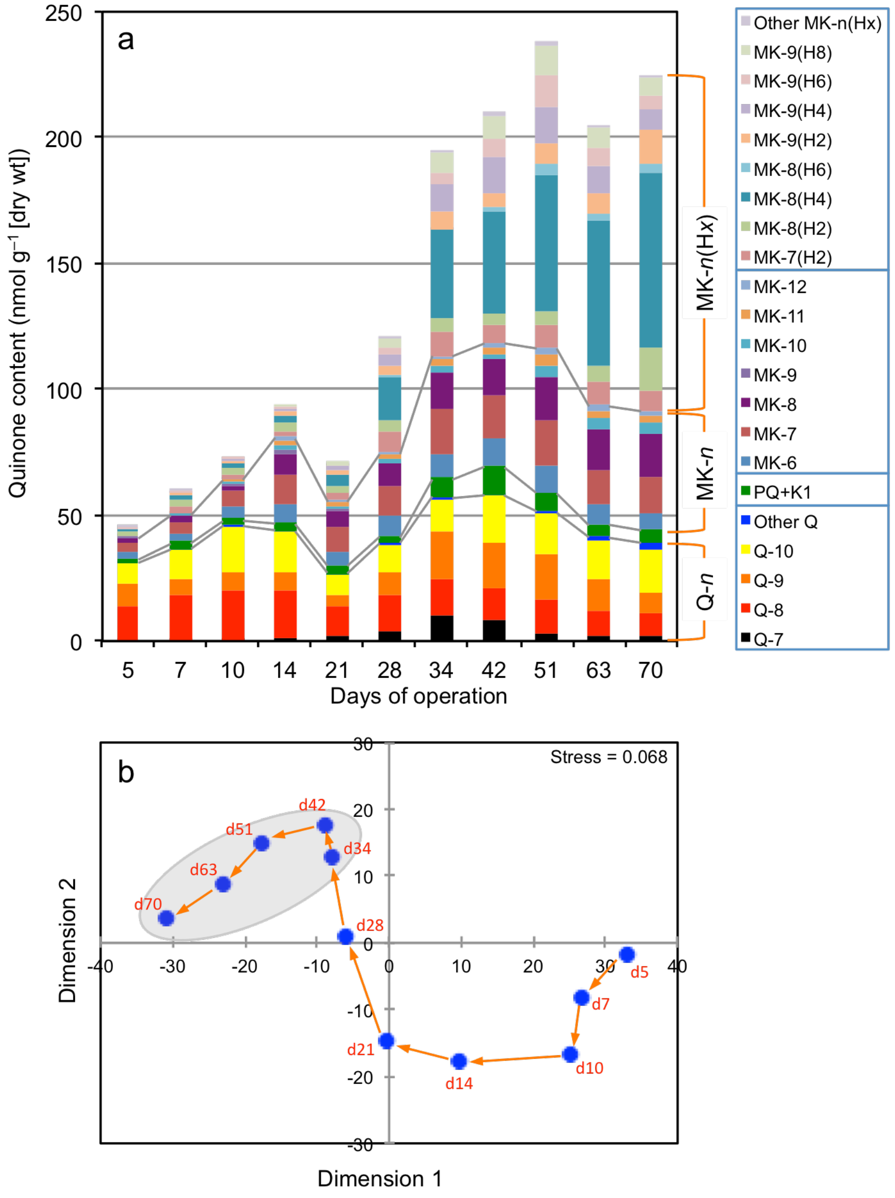

Fig. S4. Changes in quinone profiles of SCM during the overall period of FBC operation (a) and an MDS of the *D* matrix data based on the quinone profiles (b). In Fig. S4b, time-dependent shifts in quinone profiles from day 5 to day 70 (d5–d70) are shown by arrows, and a convergence of the profiles at an a_w level of less than 0.980 is surrounded by a shaded oval.

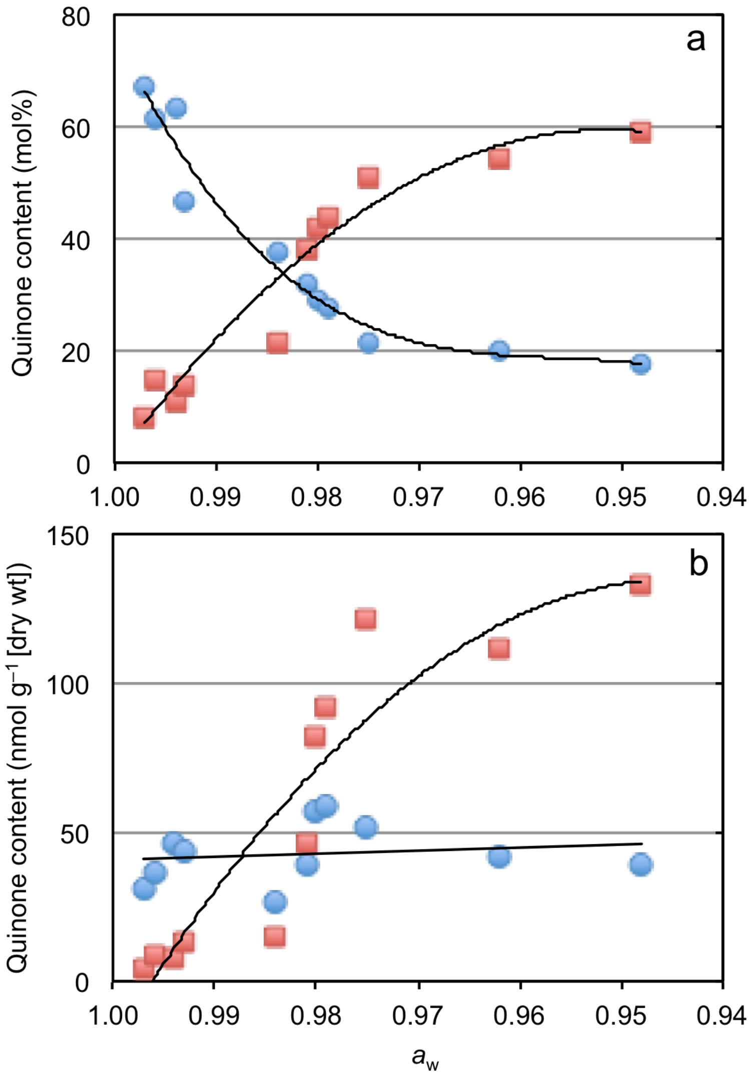

Fig. S5. Relationships between the total content (a) and relative abundance (mol%) (b) of the two quinone fractions and a_w during FBC operation. Symbols: circles, ubiquinone fraction; squares, partially hydrogenated menaquinone fraction.

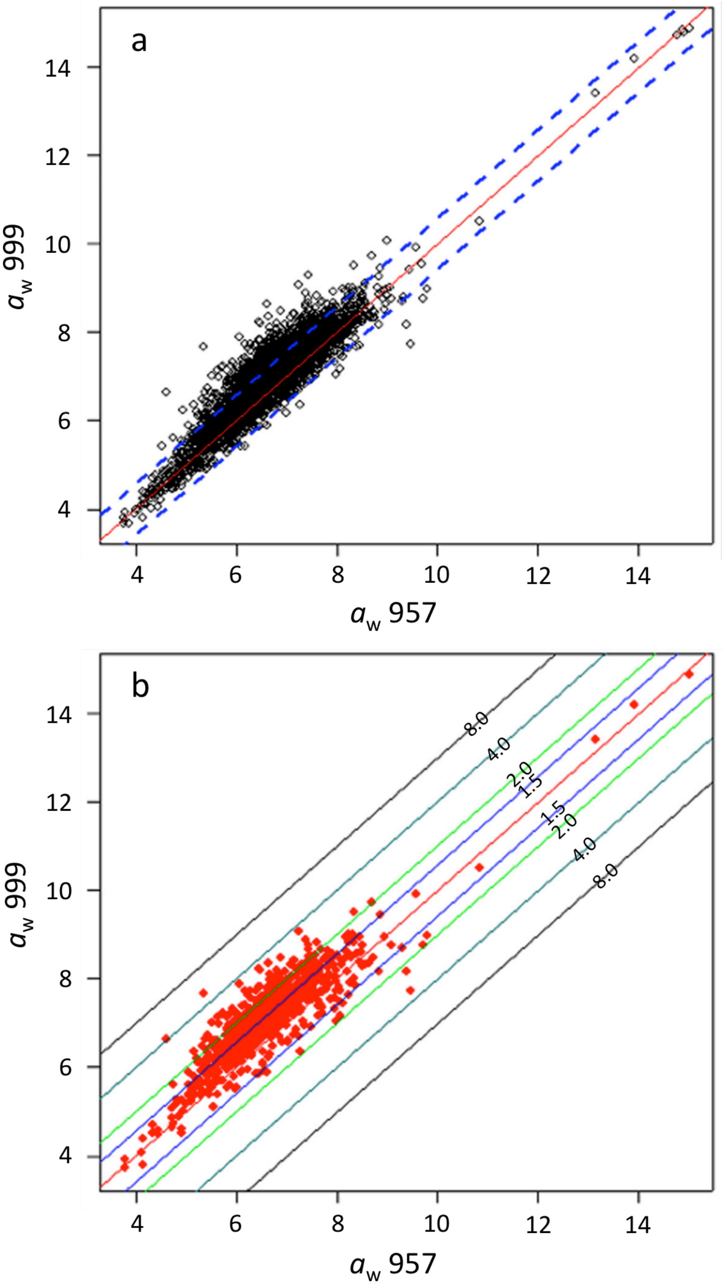

Fig. S6. Scatter plots of two microarray experiments with cDNA from *Rhodococcus* sp. strain TUT3051 cells grown at a_w 0.999 and 0.957. (a), plots of all genes expressed (R = 0.9124); (b), plots of sampled 1,005 genes whose expression was significantly different between the two by Student's t-test. The diagonal lines shows fold changes of 8.0 (black), 4.0 (blue), 2.0 (green), 1.5 (blue), and 0 ($y = x$).

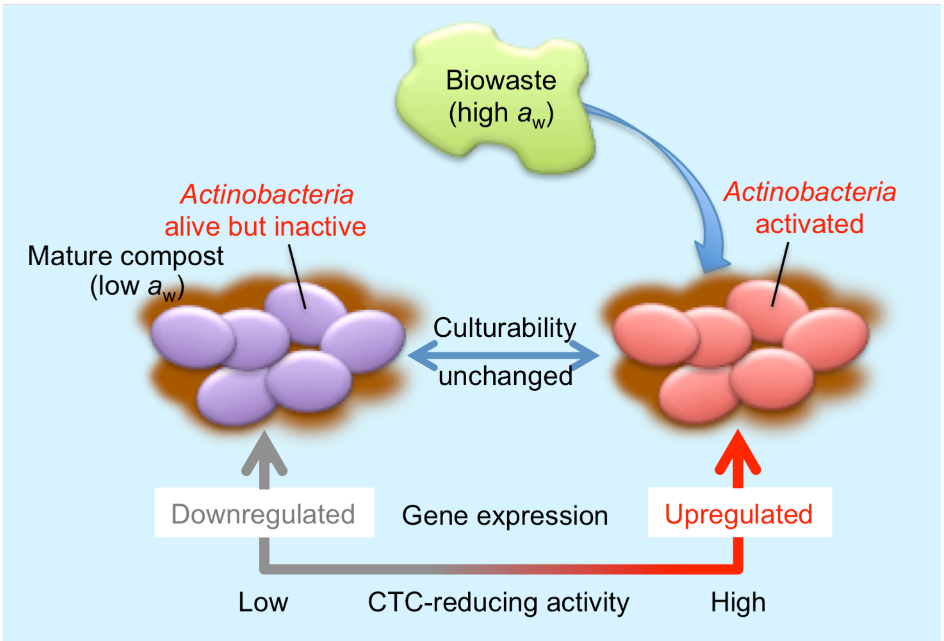

Fig. S7. Schematic model of the physiological state of *Actinobacteria* during FBC under acclimated conditions.

Table S1. Phylogenetic position and quinone systems of the aerobic chemoorganotrophic bacteria isolated from the FBC reactor on day 63

OTU no.	No. of strains	Quinone system	Closest relative based on 16S rRNA gene sequence (accession no.)[a]	Similarity (%)	Phylum/class (% of total)
1	2	Q-10	*Paracoccus* sp. TUT1021 (AB098589)	100	*Alphaproteobacteria*
2	2	Q-8	*Luteimonas* sp. TUT1238 (AB188220)	100	*Betaproteobacteria*
3	1	Q-9	*Pseudomonas* sp. TUT1023 (AB098591)	100	*Gammaproteobacteria*
4	4	MK-7	*Bacillus badius* DSM 23[T] (X77790)	99-100	*Firmicutes*
5	2	MK-7	*Bacillus* sp. TUT1206 (AB188212)	100	*Firmicutes*
6	3	MK-8	*Arthrobacter* sp. TUT1003 (AB098571)	99	*Actinobacteria*
7	2	MK-8(H_2)	*Brevibacterium luteolum* DSM 20542[T] (X77437).	100	*Actinobacteria*
8	4	MK-8(H_4)	*Cellulosimicrobium cellurans* DSM 43879[T] (X79453)	99-100	*Actinobacteria*
9	2	MK-9(H_4)	*Isoptericola* sp. TUT1252 (AB188223)	100	*Actinobacteria*
10	4	MK-8(H_4)	*Ornithinicoccus* sp. TUT1239 (AB188221)	99-100	*Actinobacteria*
11	4	MK-8(H_2)	*Rhodococcus* sp. TUT1025 (AB098593)	99	*Actinobacteria*

[a] Results of RDP Seqmatch search with an "isolates" data set option.

Table S2. Selected cDNA microarray data on differentially expressed genes in response to water activity stress

Gene or locus tag	Description of gene product	Accession no.	Fold change [a]
Carbohydrate metabolism (including TCA cycle)			
manA	Mannose-6-phosphate isomerase	NP_856928	–2.61
fucA	L-Fuculose phosphate aldolase	NP_854406	–2.08
serA2	D-3-Phosphoglycerate dehydrogenase	NP_854407	–1.79
adhB	NAD-dependent zinc-containing alcohol dehydrogenase	NP_854442	–1.77 [b]
adhE2	Zinc-dependent alcohol dehyderogenase	NP_855932	–1.74 [b]
gap	Glyceraldehyde-3-phosphate dehydrogenase	NP_855123	–1.70
icd1	Isocitrate dehydrogenase	NP_857016	–1.66
sdhC	Succinate dehydrogenase cytochrome B-556 subunit	NP_856990	–1.50
sucC	Succinyl-CoA synthetase subunit beta	NP_854633	–1.50
Energy metabolim			
nuoM	NADH dehydrogenase subunit M	NP_856826	–2.11
ndh	NADH dehydrogenase	NP_855537	–1.91
atpD	ATP synthase F0F1 subunit beta	NP_854996	–1.71
fprA	NADPH:adrenodoxin oxidoreductase FprA	NP_856778	–1.64
fdxB	Electron transfer protein FdxB	NP_857223	–1.59
cydB	Integral membrane cytochrome D ubiquinol oxidase CydB	NP_855301	–1.53
ctaE	Cytochrome C oxidase subunit III	NP_855865	–1.50
Lipid metabolism			
plsB1	Acyl transferase PlsB1	NP_855229	+1.52
tesB1	Acyl-CoA thioesterase II	NP_855297	+1.51
Mb1116A	Hypothetical protein (undecaprenyl pyrophosphate synthase)	NP_854772	–4.95
mmaA2	Methoxy mycolic acid synthase	NP_854321	–2.33
Mb3121c	Hypothetical protein Mb3121c (acyl-CoA dehydrogenase)	NP_856766	–2.11
fadD15	Long-chain-fatty-acid-CoA ligase	NP_855859	–1.54
fadD21	Acyl-CoA synthetase	NP_854871	–1.52
Nucleotide metabolism			
trxA	Thioredoxin	NP_855157	+1.50
Mb0539	Thioredoxin protein	NP_854201	–1.87
Amino acid metabolism			
asnB	Asparagine synthetase AsnB	NP_855873	–2.47
lat	L-Lysine aminotransferase	NP_856963	–1.65
glnA1	Glutamine synthetase	NP_855893	–1.51
Metabolism of cofactos and vitamins			
thiL	Thiamine-monophosphate kinase THIL	NP_856646	+1.86
ribF	Bifunctional riboflavin kinase	NP_856455	+1.51
moaD2	Molybdenum cofactor biosynthesis protein MoaD	NP_854549	+1.50
moaA	Molybdenum cofactor biosynthesis protein A	NP_854550	–2.19
coaA	Pantothenate kinase	NP_854778	–1.84
folP2	Dihydropteroate synthase 2 FolP2	NP_854893	–1.82
Cell envelope			
ponA1	Bifunctional penicillin-binding protein 1A/1B	NP_857559	+1.94
Mb3058	Glycosyltransferase	NP_856703	–2.80
wag31	Hypothetical protein Mb2169c wag31	NP_855818	–2.20 [b]
Mb1240	Glucosyl-3-phosphoglycerate synthase	NP_854894	–2.04
ftsZ	Cell division protein FtsZ	NP_855823	–1.60

[a] +, upregulated; –, downregulated.

[b] Gene expressions confirmed by proteomic analysis.

Table S2–continued

Gene or locus tag	Description of gene product	Accession no.	Fold change [a]
Transcription			
Mb3439c	Transcriptional regulator	NP_857079	+1.50
Mb0601	GntR family transcriptional regulator	NP_854261	–3.52
Mb3147	Transcriptional regulator	NP_856792	–2.44
Mb3233	TetR family transcriptional regulator	NP_856878	–2.34
sigL	RNA polymerase sigma factor SigL	NP_854414	–2.22
kdpE	Transcriptional regulator KdpE	NP_854711	–2.20
embR	Transcriptional regulator EmbR	NP_854952	–2.12
rpoB	DNA-directed RNA polymerase subunit beta	NP_854344	–2.02
Mb1078	Transcriptional repressor	NP_854733	–1.93
Translation			
rpsT	30S ribosomal protein S20	NP_857590	+1.50
aspS	Aspartyl-tRNA synthetase	NP_856248	–2.30
rpmG	50S ribosomal protein L34	NP_857590	–2.30
rpmG	50S ribosomal protein L3	NP_854739	–2.27
rpmG	50S ribosomal protein L5	NP_854359	–2.09
rpmG	50S ribosomal protein L33	NP_854311	–1.66
rpmB2	50S ribosomal protein L28	NP_855734	–1.52
Folding, sorting and degradation			
dnaJ1	Molecular chaperone DnaJ	NP_854023	–3.41
dnaK	Molecular chaperone DnaK	NP_854021	–2.98
Replication and repair			
dnaB	Replicative DNA helicase	NP_853728	+2.32 [b]
nei	Endonuclease VIII	NP_856970	–2.51
alkAb	Methylated-DNA-protein-cysteine methyltransferase	NP_855004	–1.99
recO	DNA repair protein RecO	NP_856032	–1.76
dinX	DNA polymerase IV	NP_855216	–1.65
Membrane transport			
Mb2242	Transmembrane protein	NP_855891	+1.52
lprO	Lipoprotein LPRO	NP_853850	–4.06
Mb1240	Glutamine-transport transmembrane protein ABC transporter	NP_856238	–2.04
Signal tranduction			
Mb1295	Adenyl cyclase	NP_854949	+1.55
PE/PPE family			
PE27A	PE family protein pe27a	CDO44312	+3.33
PE36	PE family protein	NP_857559	+1.93
PE13	PE family protein	NP_854881	–2.06

[a] +, upregulated; –, downregulated.

[b] Gene expressions confirmed by proteomic analysis.

Table S2–continued

Gene or locus tag	Description of gene product	Accession no.	Fold change [a]
Unknown and conserved hypotheticals			
esxD	Hypothetical protein Mb3920c	NP_857557	+1.86
Mb3703c	Hypothetical protein Mb3703c	NP_857342	+1.75
Mb2476c	Hypothetical protein Mb2476c	NP_856123	+1.65
Mb0596	Hypothetical protein Mb0596	NP_854256	+1.59
Mb2951c	Hypothetical protein Mb2951c	NP_856596	+1.58
TB27.3	Hypothetical protein Mb0592	NP_854252	+1.56
Mb3715	Hypothetical protein Mb3715	NP_857354	+1.52
Mb2757	Hypothetical protein Mb2757	NP_856403	+1.52
Mb1116A	hypothetical protein Mb1116A	NP_854772	–4.95
Mb0886	hypothetical protein Mb0886	NP_854544	–3.27

[a] +, upregulated; –, downregulated.

[b] Gene expressions confirmed by proteomic analysis.

Table S3. Identification by Muldi-TOF MS of the peptides from *Rhodococcus* sp. strain TUT3051

Spot no.	Apparent molecular size (kDa)	*m/z*	Peptide sequence	BLAST search against *Rhodococcus pyridinivorans*[a]			
				Related protein	Average MW	Accession number	Identity (%)
1	110	855.25	ELEVPVVA	DNA helicase	111,888	WP_041803920	100
		1553.96	MSEISEAPLFIDDS	Same as above			100
2	40	812.41	PLSGPR	MULTISPECIES: zinc-containing alcohol dehydrogenase [*Gordonia*][b]	39,500	NP_854442	83
		1200.60	LVLTALNR	Same as above			100
3	38	1601.68	DFPTYVDLYQQGR	Alcohol dehydrogenase	37,741	WP_024101441	100
		1737.72	DVEQAFDTMQR	Same as above			73
		1945.75	SVAVLGCNVGDAALMQR	Same as above			75
4	31	1623.74	LESQLEELEQR	MULTISPECIES: hypothetical protein [*Rhodococcus*]	30,807	WP_006554131	100
		1864.83	LKSYLESQLEELEQR	Same as above			100
5	14	920.53	HVVEALPR	MULTISPECIES: hypothetical protein [*Rhodococcus*]	15,067	WP_006552306	100
		1602.74	VADALLTVTDER	Same as above			92
		1785.89	SDALLGADQR	Same as above			70
6	9	920.45	EELFNLR	MULTISPECIES: 50S ribosomal protein L29 [*Rhodococcus*]	8,815	WP_006554603	100
		1248.65	EAKEELFNLR	Same as above			100
		1394.63	FQMATGQLNNNR	Same as above			92

[a]*R. pyridinivorans* AK37 (taxid:1114960) and *R. pyridinivorans* SB3094 (taxid: 1435356).

[b] Result of standard BLAST search.
